# Chronic non-infectious osteitis: single centre case series

**DOI:** 10.1186/1546-0096-13-S1-P184

**Published:** 2015-09-28

**Authors:** P Dawson, N Hill, M Roderick, A Finn, R Athimalaipet

**Affiliations:** 1 Bristol Children's Hospital, Bristol, UK

## Background

Chronic non-infectious osteitis (CNO), also known as Chronic Recurrent Multifocal Osteomyelitis, is a non-infectious inflammatory osteopathy predominantly affecting children and adolescents. Diagnosis is often difficult as initial symptoms and clinical course can vary widely. CNO can result in significant morbidity. Treatment regimes are varied but bisphosphonate therapy with pamidronate is proving effective at reducing pain and improving bone remodelling in some patients.

## Objective

To review the presentation, diagnosis, management and clinical course of patients with CRMO in one paediatric centre.

## Method

Case-notes of forty seven patients undergoing treatment for CNO from 2003 to 2015 at Bristol Children's Hospital were analysed and details entered into a custom-made spreadsheet containing eighty two parameters. In order to establish the criteria that had been used for diagnosis, plain films, CT, MRI, bone scans and biopsy results were recorded alongside presenting characteristics.

## Results

Median age at presentation was 10 years (range 1.5 to 14). 33 were female, 14 male. The initial differential diagnoses included Langerhans Cell histiocytosis (5), malignancy (4), infectious osteomyelitis (12), arthritis (4), musculoskeletal (5), other viral illness (11) and CNO (6). All patients had x-ray and MRI studies. Whole body STIR MRI was used to detect silent areas of osteitis and monitor treatment. The predominant sites of bony involvement on MRI included femur (12), clavicle (9), tibia (13) and spine (14). 25 patients underwent bone biopsy and all had extensive microbiological investigations, with no organisms identified. Mean time from initial presentation to medical services to diagnosis was 26 months (range 1-84). 24 of patients had been initially treated with at least one course of antibiotics for a presumed infectious osteomyelitis. Patients were also treated with NSAIDs (42), steroids (5) and DMARDs (8), although only a small proportion had pain cessation. 24 patients received pamidronate, of which 12 completed the course. Of those who completed the course 4 had persistent pain and 8 had pain cessation.

## Conclusions

This cohort is one of the largest series in the literature. It is important to increase awareness of CRMO as a diagnostic differential when a child presents with insidious onset bone pain. MRI STIR provides important evidence in the diagnosis of CRMO. A central database would facilitate a greater understanding of the diagnostic criteria and treatment options. Pamidronate has thus far appered effective in the management of 8 patients.

